# TNF blockade contributes to restore lipid oxidation during exercise in children with juvenile idiopathic arthritis

**DOI:** 10.1186/s12969-019-0354-1

**Published:** 2019-07-22

**Authors:** Emmanuelle Rochette, Pierre Bourdier, Bruno Pereira, Eric Doré, Anthony Birat, Sébastien Ratel, Stéphane Echaubard, Pascale Duché, Etienne Merlin

**Affiliations:** 1 CHU Clermont-Ferrand, Pédiatrie, Hôpital Estaing, F-63000 Clermont-Ferrand, France; 2 Université Clermont Auvergne, INSERM, CIC 1405, Unité CRECHE, F-63000 Clermont-Ferrand, France; 3 Université Clermont Auvergne, Laboratoire des Adaptations Métaboliques en conditions Physiologiques et Physiopathologiques (AME2P), EA 3533 Clermont-Ferrand, France; 4 CRNH-Auvergne, F-63000 Clermont-Ferrand, France; 5 CHU Clermont-Ferrand, Délégation de la Recherche Clinique et Innovations, F-63000 Clermont-Ferrand, France; 6 Université de Toulon, Laboratoire IAPS, F-83041 Toulon, France; 7 Université Clermont Auvergne, INRA, UMR 1019 UNH, ECREIN, F-63000 Clermont-Ferrand, France; 8 Pédiatrie, CHU Estaing, 1, place Lucie et Raymond Aubrac, 63003 Clermont-Ferrand, France

**Keywords:** Pediatric, Physical activity, Inflammation, Fat oxidation, Metabolism

## Abstract

**Background:**

Children with juvenile idiopathic arthritis (JIA) have impaired physical abilities. TNF-α plays a crucial role in this pathogenesis, but it is also involved in the use of lipids and muscle health. Objective of this study was to explore substrate oxidation and impact of TNF blockade on energy metabolism in children with JIA as compared to healthy children.

**Methods:**

Fifteen non-TNF-blockaded and 15 TNF-blockaded children with JIA and 15 healthy controls were matched by sex, age, and Tanner stage. Participants completed a submaximal incremental exercise test on ergocycle to determine fat and carbohydrate oxidation rates by indirect calorimetry.

**Results:**

The maximal fat oxidation rate during exercise was lower in JIA children untreated by TNF blockade (134.3 ± 45.2 mg.min^− 1^) when compared to the controls (225.3 ± 92.9 mg.min^− 1^, *p* = 0.007); but was higher in JIA children under TNF blockade (163.2 ± 59.0 mg.min^− 1^, *p* = 0.31) when compared to JIA children untreated by TNF blockade. At the same relative exercise intensities, there was no difference in carbohydrate oxidation rate between three groups.

**Conclusions:**

Lipid metabolism during exercise was found to be impaired in children with JIA. However, TNF treatment seems to improve the fat oxidation rate in this population.

**Trial registration:**

In ClinicalTrials.gov, reference number NCT02977416, registered on 30 November 2016.

## Background

Children with juvenile idiopathic arthritis (JIA) have impaired aerobic and anaerobic capacities [1] and reduced isometric strength [2] in both the active and inactive disease states. The impairment of their physical fitness appears to be correlated with disease severity [1]. This finding can be explained by the inactive lifestyle of JIA patients [3] due to chronic joint pain [4]. This lack of physical activity leads to increased muscle weakness and atrophy, which both alter the body composition of JIA patients, whereby their fat mass increases and their bone and lean mass decreases [5]. In addition, medications such as glucocorticoids act directly, leading to muscular loss and osteoporosis [6]. Ultimately, a vicious cycle emerges, resulting in physical deconditioning. Furthermore, physical inactivity and low-grade chronic inflammation are associated with insulin resistance, type 2 diabetes and cardiovascular diseases [7]. These co-morbidities contribute to children with JIA becoming a population at long-term cardiovascular risk.

Tumor necrosis factor alpha (TNF-α) has a crucial role in the pathogenesis of JIA, inducing expression of other pro-inflammatory cytokines such as interleukin (IL)-1β, IL-6 and IL-8, leading to an overall inflammatory response. But is also involved in tissue insulin resistance lipid utilization and muscular atrophy [8]. TNF blockade are widely indicated in the treatment of inflammatory diseases such as JIA; nevertheless, less is known about their impact on skeletal muscle metabolism, especially in children. To the best of our knowledge, only one study has investigated the effect of TNF blockade on glucose metabolism in children with JIA. This study reported no difference in plasma glucose levels before and after 3 and 6 months of TNF blockade therapy [9]. Furthermore, despite conflicting results in studies involving adults, TNF blockades have been shown to impair lipid metabolism (impaired concentrations of free fatty acids [FFAs], triglycerides, low density lipoprotein, and high density lipoprotein–cholesterol) [10]. Children treated with TNF blockade could have an energy-substrate oxidation profile during exercise which is close to healthy peers contrary to patients who are left untreated with a TNF blockade.

The two main energy sources oxidized during aerobic exercise are fats and carbohydrates (CHOs), and their relative use depends on the exercise modalities, primarily the intensity of exercise [11], but also the duration [12] and mode of exercise [13]. Some physiological variables such as sex [14], nutrient intake [15], level of physical fitness [16], and body composition—especially the fat-free mass [17] and the biological age and/or maturational status—also alter the maximal fat oxidation (MFO) rate and the mix of substrates oxidized during submaximal exercise [18, 19]. Moreover, chronic diseases such as diabetes or Crohn’s disease impact substrate oxidation during exercise, leading to markedly altered MFO values or intensity of exercise at which MFO is achieved (FATmax) with lower exercise intensities and reduced MFO rates [20, 21].

Hence, compared to healthy children, children with JIA could be characterized as having impaired oxidation of energy substrates during submaximal exercise due to a reduction in lipid oxidation. Therefore, this study compares substrate oxidation during exercise in children with JIA treated with a TNF blockade and those not treated with a TNF blockade versus healthy controls to determine: whether the pathology and TNF treatment affect these metabolic responses during submaximal exercise.

## Methods

### Patients

Thirty children (15 non-TNF blockade and 15 under TNF blockade medication) aged 7 to 18 years old with JIA (according to the International League of Associations for Rheumatology criteria) and 15 healthy controls were enrolled in the study. Participants in the three groups were matched by age, pubertal stage, and gender. All participants were followed between January 2017 and June 2019 at the pediatric unit of the Clermont-Ferrand University Hospital in France. We firstly included patients treated by TNF blockade, then we included matched JIA patients without TNF blockade and healthy controls. Healthy controls were recruited from the entourage (classmates, family, sports club) of the patients.

Participants were excluded if they had a physician-diagnosed infection, had received oral corticosteroids within the previous 3 months, had chronic disease (other than JIA for patients). All treatments had been administered for at least 3 months at the time of evaluation. Disease status (active or inactive) was evaluated according to the American College of Rheumatology criteria [22]. The physical activity level (PAL) was determined according to the International Physical Activity Questionnaire for Adolescents (IPAQ-A), and individuals were classified into one of three levels of physical activity (PA): low, moderate, or high.

The study was approved by the governing ethics committee (Comité de Protection des Personnes [CPP] Sud-Est VI – Clinical trial number NCT 02977416). All participants and their parents gave their informed consent and were free to withdraw from the study at any time.

### Experimental procedure

Participants were asked to refrain from consuming any food or liquid, with the exception of water, 3 h before the visit. Participants were in minimal fasting state of 3 h. They also avoided high fat food and refrained from strenuous physical activity for at least 24 h before the visit. Participants arrived the hospital laboratory for indirect calorimetry test in the morning. After sitting quietly for 20 min, participants performed a graded exercise test to volitional fatigue on an electromagnetically-braked cycle ergometer with continuous gas collection and heart rate monitoring. Following a 2-min warm-up involving unloaded pedaling, Tanner stages 1 and 2 participants started cycling at 10 W and the work rate was increased by 10 W every 3 min. Tanner stages 3 and 4 participants started at 20 W and the work rate was increased by 15 W every 3 min. In cases where the heart rate was unstable, this stage was extended for up to 5 min to obtain a stable heart rate of ±5 beats. When the respiratory exchange ratio (RER) was ≥1.00—indicating the absence of fat oxidation—, the work rate was increased by the same increments at 1-min intervals until volitional fatigue was reached. The VO_2peak_ was considered to have been reached when the RER was ≥1.05 and the participant achieved his or her age-predicted maximal heart rate (HR_max_: 220 − age), according to the methodology validated by Riddell et al. [23].

### Measurements

All the tests were performed on a Cyclus 2 ergometer (RBM Elektronik-Automation GmbH, Leipzig, Germany). The O_2_ consumption (VO_2_) and CO_2_ production (VCO_2_) were measured breath-by-breath through a mask connected to an O_2_ and CO_2_ analyzer (MetaMax 3b, Cortex Biophysik, Leipzig, Germany).

Ventilatory parameters were averaged every 1 min during the submaximal exercise test and during the subsequent 10-min recovery period. The heart rate was continuously monitored over the duration of the tests (Polar RS800cx monitor, Polar, Finland).

### Data analysis

Indirect calorimetry is known to be the standard method to quantify substrate oxidation rates at rest and during exercise [24]. The VO_2_ and VCO_2_ values were averaged over the last minute of each work rate, with the results then being used to calculate fat oxidation over a wide range of exercise intensities for each participant [25] by employing Péronnet and Massicotte’s equation [26]. Lipids (g.min^− 1^) = 1.6946 x VO_2_–1.7012 x VCO_2_ and CHO (g.min^− 1^) = 4.585 VCO_2_–3.2255 VO_2_.

For each individual, a best-fit polynomial curve was constructed for their fat and CHO oxidation rate (expressed as mg.min^− 1^) vs. exercise intensity (expressed as % VO_2peak_). Each individual curve was then used to determine the peak fat oxidation rate (MFO) and the exercise intensity that was associated with the Fatmax [25].

### Statistical considerations

The sample size estimation was determined according to previous works reported in the literature (Nguyen et al. with 11 patients by group [21]) and for an expected effect size of around 1 (as the standard deviation of the percentage change in the maximum rate of lipid oxidation ranged between 1 and 2.2). For a two-sided type-I error of 1.8% (the correction required due to multiple comparisons) and for a statistical power greater than 80%, it was proposed that 22 participants should be included in each group. Furthermore, due to the lack of data in the literature concerning (1) between- and within- participant variability and (2) the expected differences due to TNF blockade use, an exploratory interim statistical analysis was planned 15 participants by group, which allow to highlight an effect-size greater than 1.2 with aforementioned statistical assumptions.

Statistical analyses were performed using Stata software version 13 (StataCorp, College Station, TX). Tests were two-sided with the type-I error set at *α* = 0.05. Continuous data were expressed as the mean ± standard deviation (SD) or as the median (interquartile range) according to the statistical distribution (the assumption of normality was assessed with the Shapiro–Wilk test). To account for the between- and within-patient variability due to several measures being taken for the same participant, random-effects models for the correlated data were undertaken rather than the usual statistical tests, as they would have been inappropriate due to an unverified assumption of independence. Time-point evaluations, groups (with/without a TNF blockade and controls) and their interactions were considered as fixed effects, whereas the participant (patient) was considered as a random effect (slope and intercept). A Sidak’s post hoc test for multiple comparisons was applied. The normality of the residuals from these models was studied as described above using the Shapiro–Wilk test. When appropriate, the data were log-transformed to achieve normality of the dependent endpoint. Concerning non-repeated measures, quantitative variables (notably age, body mass index [BMI], disease duration, VO_2peak_, VO_2peak_.kg^− 1^ of body weight, MFO, physical activity level) were compared between groups by ANOVA or by using the Kruskal–Wallis test when assumptions required for the ANOVA were not met (normality and homoscedasticity as analyzed via the Bartlett test). When appropriate (omnibus *p*-value < 0.05), a post hoc test to account for multiple comparisons was performed: A Tukey–Kramer post-ANOVA and a Dunn test after the Kruskal–Wallis test. As less than 5% of the data were missing, the missing data were not dealt with.

## Results

### Participant characteristics

The characteristics of the participants are given in Table 1. No significant difference were observed for age, BMI, VO_2peak_, VO_2peak_ (/kg of body mass), disease activity, physical activity levels, and resting metabolism between the three groups.

**Table 1:** Participant characteristics

	JIA with TNF-α blockade	JIA without TNF-α blockade	Healthy controls	*p*
n	15	15	15	
Sex (n; Male/Female)	2/13	2/13	2/13	
Age (years) mean ± SD	13.5 ± 2.2	13.6 ± 2.8	13.7 ± 2.7	0.91
Tanner stage (n; I–II / III–IV)	4/11	4/11	4/11	
Body mass (kg) mean ± SD	49.3 ± 16.3	46.0 ± 13.5	48.9 ± 13.5	0.78
Height (cm) mean ± SD	155.8 ± 15.5	155.5 ± 14.6	154.9 ± 13.2	0.98
BMI (kg/m^2^) mean ± SD	19.6 ± 4.1	18.6 ± 3.0	20.0 ± 3.4	0.49
JIA subtype (n)			–	
oJIA	3	7	–	
pJIA RF−	7	4	–	
pJIA RF+	2	1	–	
ERA	2	1	–	
Psoriatic	1	1	–	
Undifferentiated	0	1	–	
Disease duration (months) mean ± SD	48.1 ± 31.6	46.7 ± 34.1	–	0.61
Disease activity ^a^ (n)			–	
Active	4 ^b^	4 ^c^	–	0.66
Inactive	11	11	–	
DMARDs (n)			–	
NSAIDs	1	4	–	
MTX	8	8	–	
TNF-α blockade	15	0	–	
Adalimumab	3	0	–	
Etanercept	10	0	–	
Infliximab	2	0	–	
IPAQ score (n)				
Low level of activity	8	6	5	0.69
Moderate level of activity	3	5	7	
High level of activity	4	4	3	
VO_2peak_ (ml/min) mean ± SD	1568.8 ± 670.9	1464.9 ± 445.3	1637.3 ± 579.9	0.76
VO_2peak_/body mass (ml/kg/min) mean ± SD	33.0 ± 9.9	32.6 ± 6.0	33.7 ± 8.0	0.83
Rest metabolism (kcal/day) mean ± SD	1467.2 ± 427.6	1431.7 ± 423.7	1767.6 ± 622.4	0.14

*JIA* juvenile idiopathic arthritis, *oJIA* oligoarticular JIA, *pJIA RF−* rheumatoid factor-negative (RF−) polyarticular JIA, *pJIA RF+* rheumatoid factor-positive (RF+) polyarticular JIA, *ERA* enthesitis-related arthritis, *psoriatic* psoriatic JIA, *DMARDs* disease-modifying antirheumatic drugs, *MTX* methotrexate, *NSAIDs* nonsteroidal anti-inflammatory drugs, *IPAQ* International Physical Activity Questionnaire. ^a^ according to Wallace et al. 2011; ^b^ 1 pJIA RF-, 1 pJIA RF+, 1 psoriatic, 1 ERA; ^c^ 2 oJIA, pJIA RF-, 1 pJIA RF+

In JIA patients, the disease duration was the same in the treated and untreated TNF blockade groups (48.1 ± 31.5 months vs. 46.7 ± 34.1, *p* = 0.61). Five patients received no medication, two were treated only with nonsteroidal anti-inflammatory drugs (NSAIDs), two were treated with methotrexate (MTX) plus NSAIDs, six were treated only with MTX, two with MTX plus adalimumab, four with etanercept, six with MTX plus etanercept, two with infliximab, and one was treated with adalimumab plus NSAIDs. The mean duration of the TNF blockade treatment was 25.8 ± 19.1 months.

### Fat and carbohydrates (CHO) oxidation rates

**Fig. 1:**
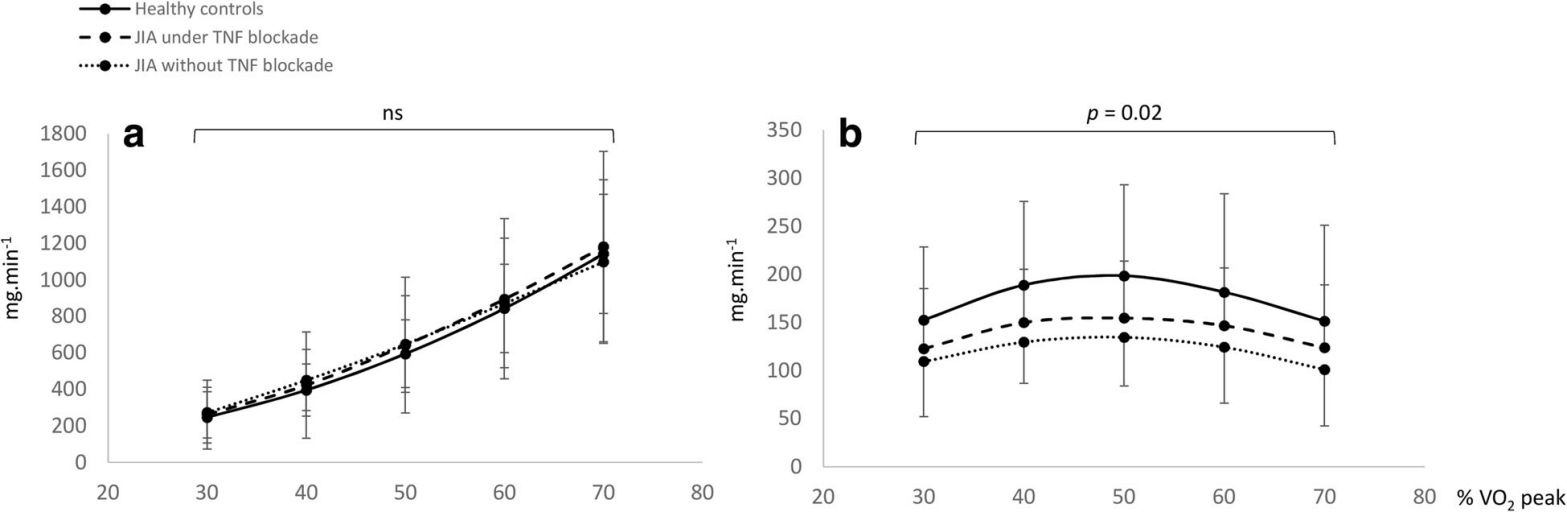
Comparison of CHO (**a**) and fat (**b**) oxidation rates from 30 to 70% of the VO_2peak_. Data are means ± SD. Significantly different at *p* < 0.05 for a comparison between JIA children without a TNF-α blockade vs. healthy controls

The fat and CHO oxidation rates relative to exercise intensities are displayed in Fig. 1a and b. The CHO oxidation rates were the same for each group at the same relative exercise intensities (Fig. 1a). However, lipid oxidation rates were statistically lower in JIA children without TNF blockade than controls from the exercise intensities corresponding to 30% of VO_2_ peak up to those corresponding to 70% of VO_2_ peak (*p* = 0.02).

At the relative exercise intensity of 30 to 70% of the VO_2peak_, fat oxidation rate was higher in the healthy controls than JIA children treated with TNF blockade (*p* = 0.14) (Fig. 1b).

### MFO

**Fig. 2:**
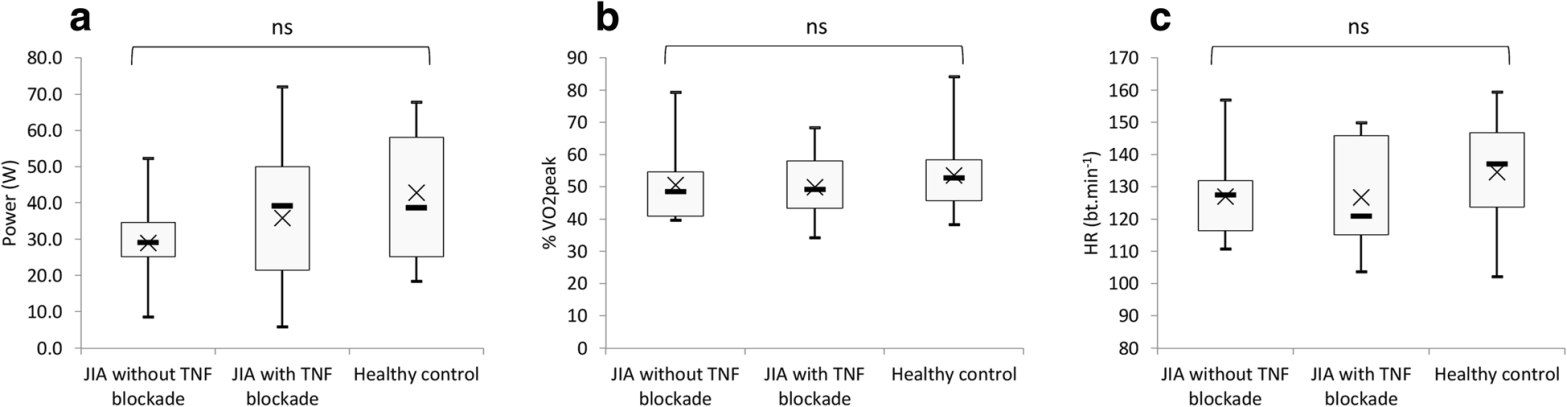
Comparison of power (**a**), % VO_2peak_ (**b**), and heart rate (**c**) at the MFO. Boxes represent interquartile ranges and whiskers give minimum and maximum values. Data are means (x) and medians (−)

The heart rate, power, and %VO_2peak_ at which the MFO for each group was reached are shown in Fig. 2. MFO was reached at a power of 42.8 ± 17.6, 35.9 ± 20.3, and 29.0 ± 11.2 W (*p* = 0.19) in the healthy controls, JIA children with TNF blockade and children JIA without TNF blockade, respectively. The heart rate reached at MFO was 135 ± 18, 127 ± 17, and 127 ± 13 beat.min^− 1^ in the healthy controls, JIA with TNF blockade and JIA without TNF blockade, respectively (*p* = 0.40). Furthermore, MFO was reached at 53.6 ± 12.3, 49.9 ± 9.9, and 50.6 ± 11.5 of the %VO_2peak_ in healthy controls, JIA with TNF blockade and JIA without TNF blockade respectively (*p* = 0.67).

**Fig. 3:**
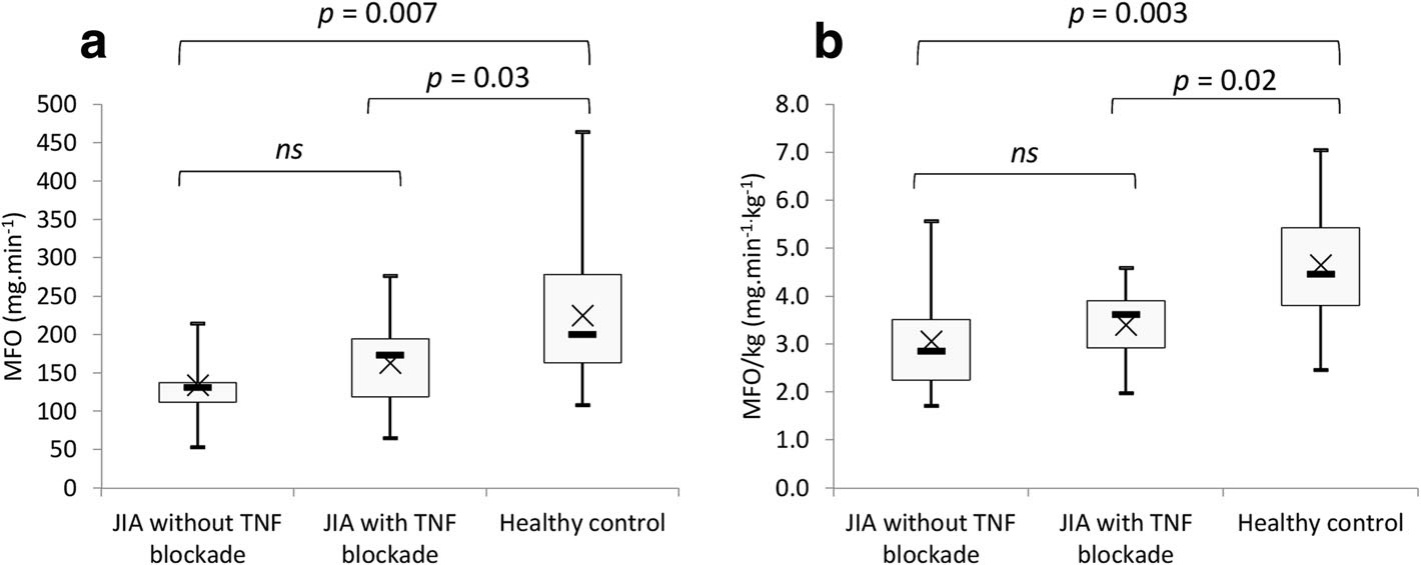
Comparison of maximal fat oxidation (**a**) and maximal fat oxidation per kg of total body mass (**b**). Boxes represent interquartile ranges and whiskers give minimum and maximum values. Data are means (x) and medians. ns: not significantly different

Maximal fat oxidation rates expressed in mg.min^− 1^ and relative to total body mass (Fig. 3) were higher in healthy controls than JIA with TNF blockade and JIA without TNF blockade (225.3 ± 92.9, 163.2 ± 59.0, 134.3 ± 45.2 mg.min^− 1^, *p* = 0.008 and 4.7 ± 1.8, 3.4 ± 0.8, 3.1 ± 1.2 mg.min^− 1^.kg^− 1^, *p* = 0.003, respectively).

## Discussion

The first main finding from the present study is that during submaximal exercise, the fat oxidation rate was higher in healthy controls than in JIA patients. Secondly patients under TNF blockade had an intermediate fat oxidation rate profile that was higher than in JIA untreated by TNF blockade but lower than in the healthy controls. These results indicate an apparent dysregulation of fat metabolism during exercise in JIA patients that improves with TNF blockade treatment.

The VO_2peak_ per total body mass values were comparable in the three groups, suggesting that the JIA children did not exhibit cardiopulmonary deconditioning in comparison to the controls. Our maximal fat oxidation rates were consistent with other studies in healthy children, although our values in mg.min^− 1^.kg^− 1^ were found to be lower since they were normalized to the total body mass and not to total lean mass [12]. The values for the maximal fat oxidation rate were lower in JIA patients than in the controls, illustrating a metabolic impairment during exercise. Given the small number of patients in each subtype, we cannot do analysis according to all subtypes of JIA. However, when we look at each subject according to their controls, the fat oxidation rate or maximal fat oxidation rate (MFO) of JIA patients are always lower than those of controls. It is also important to note that even in children with an inactive disease, the lipid oxidation rate remains lower than that of the controls, which could be a reflection of persistent subclinical inflammation. Which would mean that the criteria for defining an inactive disease are not optimal. As defined by Wallace et al. [22], the criteria for an inactive disease only use one biological criterion (C-reactive protein (CRP) or erythrocyte sedimentation rate (ESR) level within normal limits) to define the activity of the disease; other biological biomarkers (such as inflammatory cytokine levels), might thus be relevant in the absence of clinical signs [27].

Metabolic flexibility is the ability of the skeletal muscle to adapt its fuel preference to substrate availability and to its energy requirements [28]. Substrate availability is a contributor to fat oxidation during submaximal exercise, especially the FFA concentration [29]. During low-intensity exercise (from 25 to 40% of the VO_2peak_), the rate of plasma FFA uptake is sufficient to account for most of the fat metabolism. However, during strenuous work, there is a greater contribution from muscle triglycerides to total fat oxidation [29]. Furthermore, one study reported that after 12 months of treatment with etanercept, the triglyceride level increased in JIA patients compared to their baseline level prior to starting treatment [30]. Our results show that JIA patients oxidize less lipids than the controls. This means that children with JIA have impaired metabolic flexibility, yet this could to be improved with TNF blockading. The main site where the adaptive responses to exercise occur in terms of metabolic flexibility is in skeletal muscle [28]. An impairment in substrate uptake, transportation, or oxidation within the skeletal muscle can also affect the fat oxidation rate and thus could explain our results. Especially the differences in lipid oxidation at higher exercise intensities between the JIA patients and controls suggests that the substrate-metabolism impairment is at the muscle level.

TNF-α may influence skeletal muscle metabolism via different actions. First, TNF-α is known to stimulate muscle catabolism and alter contractile function in inflammatory diseases [31]. It could be assumed that children with JIA have a lower muscle mass than healthy controls or defect in motor unit recruitment, which could explain their lower absolute rate of lipid oxidation.

However, as there is no difference in the carbohydrate oxidation rate, it does not seem to be a quantitative but rather a qualitative muscle impairment. Skeletal muscle is an insulin-sensitive organ that plays a crucial role in maintaining systemic glucose homeostasis [32]. Inflammation and insulin resistance are closely related and inflammatory cytokines such as TNF-α, interleukin (IL)-6, IL-1, and IL-8 may inhibit insulin signaling via multiple mechanisms [33]. TNF-α induces the phosphorylation of insulin receptor substrate 1 (IRS-1) on serine instead of tyrosine residues, promoting insulin resistance [34]. This phosphorylation of the serine residues halts the physiological activation of the receptor, thus stopping the insulin signal [35]. In adults with rheumatoid arthritis, several studies have shown that anti-TNF therapy increases insulin sensitivity [36]. It appears that anti-TNF antibodies restore the phosphorylation status of Ser312-IRS-1 and protein kinase B (Akt), which are important mediators of the insulin-signaling cascade [36]. Moreover, another TNF-α pathway that could be implicated is the inhibition of adenosine monophosphate-activated protein kinase (AMPK) by TNF-α. AMPK inhibition by TNF reduces the mitochondrial transportation of FAs for β-oxidation [37]. All these mechanisms may explain the higher rate of lipid oxidation in our TNF blockaded JIA children, since improvements in insulin sensitivity are associated with enhanced rates of fat oxidation [38].

In our study, a higher MFO was observed in healthy controls when compared with JIA groups. Moreover, JIA with TNF blockade presented a higher MFO when compared with JIA without TNF blockade, although FATmax (intensity of exercise at which MFO is achieved) was similar between JIA groups and also between healthy controls and JIA groups. A high level of interindividual variation exists in both maximal rates of fat oxidation and the intensity at which maximal rates of fat oxidation occur (FATmax) [16]. But also, exercise training leads to a higher MFO although not necessarily to a higher FATmax [16]. It is therefore necessary to explore what explains the difference between MFO and FATmax observed in healthy children and JIA treated or not with anti-TNF-α (physical level, inflammation, treatment). Impaired lipid metabolism may be the whole body’s adaptation to a state of less activity and low energy expenditure resulting from the low level of physical activity of children with JIA [39, 40]. Lipid oxidation capacity is related to physical fitness, itself related to levels of physical activity. But, since we matched the physical activity levels of our patients with JIA to those of controls, this impairment is likely due to the disease.

Finally, hormones can influence the lipid oxidation rate and the balance of substrates during exercise. For instance, leptin activates AMPK and increases fat oxidation within the skeletal muscle [41]. Interestingly, one study reported that JIA patients had higher serum levels of leptin than the controls did, and 43 of the 49 patients in the study were undergoing biological therapy (etanercept, adalimumab, or infliximab) [42].

Our results show that, there is a qualitative alteration of muscle metabolism in JIA. This could have long-term repercussions on health, especially in a cardiovascular point of view. Since lower MFO may be associated with lower insulin sensitivity and, thus, potentially CVD risk on the long term [43].

The relatively small number of patients could be considered as a limitation. A second limitation of this study is the heterogeneity of our patient group. However, we matched patients by pubertal status, gender, and level of physical activity to reduce any bias. In addition, in future studies, the evaluation of their body composition should be taken into account (especially their lean body mass) as well as measurements of their blood lipid, glucose, and insulin levels. In addition, the population under study had different impact diseases (active or inactive disease i.e. without clinical manifestation according to Wallace et al., 2011 [22]). Whether our results can be transposed to children with active or inactive diseases is open to debate. Nevertheless, further research is warranted with a more homogenous population to confirm our results and evaluate the impact of the activity of the disease or of other treatments (methotrexate or IL-6 blockade for example). Finally, although our results showed an improvement in fat metabolism under TNF blockade in children with JIA, the oxidation rates remained lower than those observed in healthy children. We cannot exclude that these children with arthritis have painful phases that make them less active in their everyday lives in terms of physical activity. Any assessment via actimetry to assess their levels of physical activity would be required to determine their spontaneous activity.

## Conclusion

Juvenile idiopathic arthritis was found to be associated with a metabolic dysregulation of the fat oxidation rate during submaximal exercise, also in an inactive disease (without clinical manifestation) with lower maximal fat oxidation as compared to healthy peers. When children were treated by TNF blockade, fat oxidation rate during submaximal exercise was improved. This metabolic involvement could result from infra-clinical inflammation in JIA patients, which seems to be more controlled by TNF blockade treatment. Impaired metabolic control of lipid is associated with overweight and cardiometabolic risk, but lipid oxidation capacity can be improve by regular physical activity. Whether such alterations can be reversed by training needs to be assessed.

## Data Availability

The datasets used are available from the corresponding author on reasonable request.

## Abbreviations

Akt: Protein kinase B
AMPK: Adenosine monophosphate-activated protein kinase
BMI: Body mass index
CHO: Carbohydrates
CPP: Comité de Protection des Personnes
CVD: Cardiovascular disease
Fatmax: Intensity of exercise at which MFO is achieved
FFAs: Free fatty acids
IL: Interleukin
IRS-1: Insulin receptor substrate 1
JIA: Juvenile idiopathic arthritis;
MFO: Maximal fat oxidation
MTX: Methotrexate
NSAIDs: Nonsteroidal anti-inflammatory drugs
PA: Physical activity
PAL: Physical activity level
RER: Respiratory exchange ratio
SD: Standard deviation
TNF-α: Tumor necrosis factor alpha;
VCO_2_: Carbon dioxide volume
VO_2_: Oxygen volume
W: Watt
